# A complex case of chronic cutaneous *Borrelia burgdorferi* sensu stricto infection manifesting as morphea-like sclerosis in the United States

**DOI:** 10.1016/j.jdcr.2026.01.038

**Published:** 2026-02-02

**Authors:** Marisa R. Diiorio, Guohong Huang, Payal C. Shah, Shaofeng Yan, Robert E. LeBlanc, Joel A. Lefferts, Lin A. Brown, Dorothea T. Barton

**Affiliations:** a Geisel School of Medicine, Hanover, New Hampshire; b Department of Pathology and Laboratory Medicine, Dartmouth Hitchcock Medical Center, Lebanon, New Hampshire; c Department of Dermatology, Dartmouth Hitchcock Medical Center, Lebanon, New Hampshire; d Department of Rheumatology, Dartmouth Hitchcock Medical Center, Lebanon, New Hampshire

**Keywords:** acrodermatitis chronica atrophicans (ACA), *Borrelia burgdorferi* (*B. burgdorferi*), droplet digital polymerase chain reaction (ddPCR), fibrosing Borrelia-associated dermatosis, Lyme disease, morphea

## Introduction

Lyme disease presents with a broad spectrum of cutaneous manifestations, most commonly being erythema migrans, Borrelial lymphocytoma, and acrodermatitis chronica atrophicans (ACA).^1^ In the United States (U.S.), where *Borrelia burgdorferi* sensu stricto (*B. burgdorferi* s.s.) predominates, erythema migrans is the most prevalent and presents as an expanding erythematous patch or plaque in early localized infection.^1^ In contrast, ACA is a late, chronic manifestation linked to European strains, particularly *B. afzelii*.^2^ ACA begins as swollen violaceous plaques on distal extensor surfaces that may progress to thin, atrophic skin, and includes peripheral neuropathy, arthropathy, or nodules over bony prominences in some cases.^1^^,^^2^

Beyond these entities, *B. burgdorferi* has been implicated in fibrosing dermatoses such as morphea and reactive granulomatous dermatitis, which can overlap clinically and histopathologically with ACA.^3^, ^4^, ^5^, ^6^ ACA reflects an infection-driven process, whereas morphea is an autoimmune sclerosing disorder, often associated with autoantibodies and triggered by trauma, vaccination, or infection.^4^^,^^7^ It is typically managed with long-term immunomodulation.^4^^,^^7^ Morphea has many subtypes, including generalized, linear, acral, profunda, and the rare nodular variant.^7^^,^^8^

Here, we report a diagnostically complex and uncertain case of a chronic fibrosing *Borrelia* dermatosis, confirmed by droplet digital polymerase chain reaction detection of *B. burgdorferi* s.s. in lesional tissue, presenting with morphea-like sclerosis and rapid, complete response to doxycycline.

## Case summary

**Fig 1:**
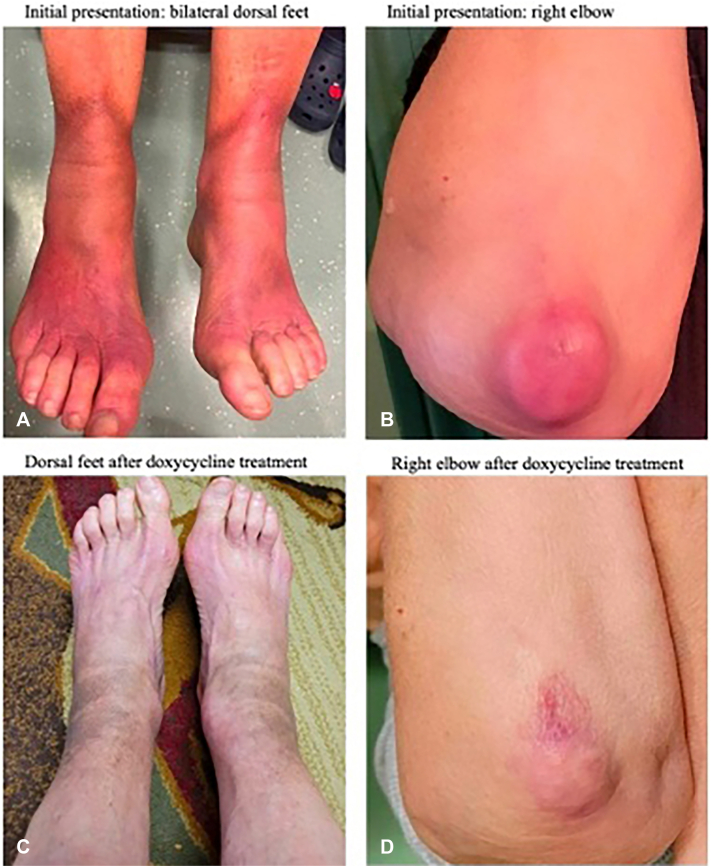
Parts **A-D**, Clinical presentation before and after treatment with doxycycline.

A 73-year-old woman presented with a 4-year history of progressive induration, pain, and tingling around the ankles, leading to limited joint mobility and difficulty ambulating. Prior courses of systemic corticosteroids provided minimal benefit. Examination revealed well-demarcated violaceous to red-brown indurated plaques on the dorsal feet and ankles with firm “step-offs” from uninvolved skin (Fig 1, *A*). Indurated nodules over bony prominences were seen on the mid-spine, bilateral knees, and right elbow (Fig 1, *B*).

**Fig 2:**
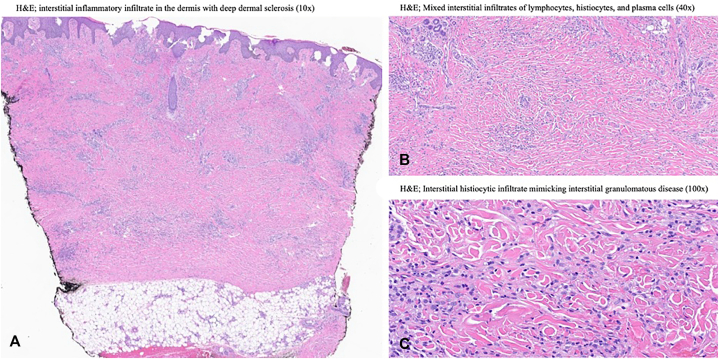
Parts **A-C**, Histopathology slides of multiple skin biopsies with hematoxylin and eosin (H&E) staining.

Laboratory studies showed a mildly elevated erythrocyte sedimentation rate and C-reactive protein, positive antinuclear antibody at 1:320, positive anti-*B. burgdorferi* IgG with negative IgM titers, and a negative extractable nuclear antigen panel. Skin biopsies revealed superficial and deep perivascular and interstitial inflammatory infiltrates with deep dermal sclerosis (Fig 2, *A*) and decreased dermal CD34 stromal cell staining. The presence of mixed interstitial infiltrates of lymphocytes, histiocytes, and plasma cells were visualized extending into the subcutis (Fig 2, *B* and *C*).

**Fig 3:**
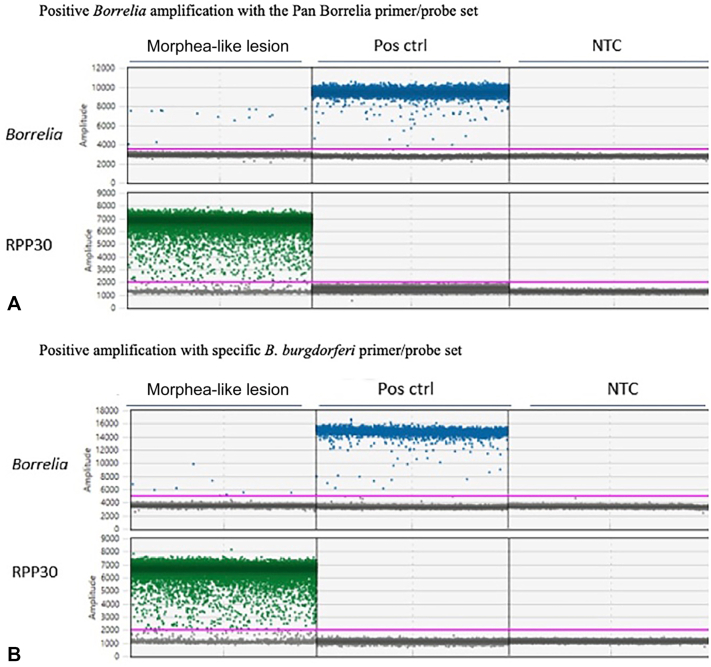
Parts **A,** and **B,** Positive *Borrelia* amplification with the Pan Borrelia primer/probe set. *NTC*, nontemplate control.

Given the positive anti-*Borrelia* serologies without a known history of Lyme disease or prior antibiotic treatment, a therapeutic trial of doxycycline (100 mg twice daily for 30 days) was provided. This resulted in rapid, near-complete resolution of the plaques with restoration of joint mobility (Fig 1, *C* and *D*). Total DNA extracted from formalin-fixed, paraffin-embedded lesional tissue was analyzed using droplet digital polymerase chain reaction. Positive droplets using *B. burgdorferi*-specific primer/probe sets were detected (Fig 3, *A* and *B*), confirming *B. burgdorferi* s.s. as the causative species. The droplet digital polymerase chain reaction absolute quantification profile was consistent with only *B. burgdorferi,* which was further confirmed by Sanger sequencing.

The RPP30 gene served as an internal control, and it was targeted with a commercial primer/probe mix from Bio-Rad (Cat # 10031243). (A) Pan Borrelia primer/probe set,^6^ targeting the 5S-23S intergenic spacer region: 5′-TTCTTCGCCTTAAAGCTCCT-3′ (Forward), 5′-TGGCAAAATAGAGATGGAAGAT-3′ (Reverse), 5′-FAM-ATTACTTTGACCATATTT-MGBNFQ-3′ (Probe) (B) B. burgdorferi-specific primer/probe set, targeting the ospA gene: 5′-TTGAAGGCGTAAAAGCTGAC-3′ (Forward), 5′-ACTAGTGTTTTGCCATCTTC-3′ (Reverse), 5′-FAM-TCTGACGATCTAGGTCAAACCACACT-BHQ1-3′ (Probe). Pos ctrl: positive control. NTC: nontemplate control.

## Discussion

We present a rare and diagnostically uncertain case of chronic fibrosing *Borrelia*-associated dermatosis. ACA and morphea were both strongly considered given overlapping clinical, histopathologic, and molecular features; however, inconsistencies with each entity prevented a unifying diagnosis.

Several aspects of our patient’s presentation overlap with features reported in the largest and most comprehensive study of ACA to date,^2^ including bilateral acral violaceous plaques of the lower-extremities and nodules on extensor elbows, both with lesional molecular confirmation of *B.burgdorferi* s.s.,^2^^,^^3^^,^^6^ as well as neuropathic symptoms confined to affected joints.^2^ However, important inconsistencies exist. Notably, the lesions were sharply well-demarcated, favoring morphea,^7^ rather than the ill-defined, progressive plaques typical of ACA.^2^ Additionally, *B. burgdorferi s.s.* is documented to be a rare cause of ACA, and there was no clinical or histopathologic evidence of cutaneous atrophy. In the medical literature, most but not all patients (59%) exhibit at least 1 atrophic feature at presentation, with a median progression time of 12 months from inflammatory to atrophic stages, and longer symptom duration correlating with an increased likelihood of atrophy.^2^ In this context, a 4-year disease course without atrophic progression adds further uncertainty to ACA as a unifying diagnosis. Given *B. burgdorferi’s* association with more atypical ACA features such as arthropathy, it remains plausible that *B. burgdorferi* infection may incite a more inflammatory phenotype or slower progression to atrophic stages compared with *B. afzelii,* but this requires further formal investigation.^2^

*B. burgdorferi-associated morphea* was also carefully considered for diagnosis.^4^ The deeply indurated, thickened, well-demarcated plaques without atrophy, along with joint restriction, ANA positivity, and certain histological features (Fig 2, *A*), could support an autoimmune sclerosing process consistent with morphea.^5^^,^^7^^,^^8^ As often seen with morphea, corticosteroids were ineffective in this patient. Atypically, however, doxycycline achieved rapid and near-complete resolution. While some European reports describe antibiotic-responsive *Borrelia*-associated morphea, it remains unclear whether these represent primary morphea or *Borrelia* infection triggering a reactive sclerosing dermatosis.^3^^,^^5^ Dermatology literature more generally indicates that effective management of primary morphea requires long-term immunosuppression and often has minimal response to antibiotics.^3^^,^^7^ The presence of acral nodules is also atypical,^7^ and the concurrence of 2 rare morphologic variants (acral and nodular) in a single patient, together with near-complete antibiotic response, contributes to the uncertainty of primary morphea as a unifying diagnosis.^7^^,^^9^ However, *Borrelia* infection triggering reactive morphea may still be possible.

Histopathologic findings further support an infection-driven process. Sclerosing features seen in morphea are present in this patient (Fig 2, *A*); however, the RGD-like patterns and subcutaneous involvement also seen here (Fig 2, *B* and *C*), are well-documented in *Borrelia*-associated dermatoses, but are not characteristic of morphea.^6^ Loss of CD34 staining, often cited in morphea, is a nonspecific feature and also occurs in chronic *Borrelia*-related sclerosis, including ACA.^6^ Collectively, these histopathologic findings evidence *B.burgdorferi* infection inducing sclerotic, atrophic, and granulomatous microscopic changes, and underscores the diagnostic complexity of this case.^3^^,^^6^

This case offers a rare, molecularly confirmed example of a chronic cutaneous fibrosing Lyme borreliosis in the U.S., demonstrating both pathogen detection and rapid antibiotic response. This reinforces the importance of early serologic and molecular *Borrelia* testing in sclerosing dermatoses, particularly in endemic regions, before initiating immunosuppressive therapy, which may be avoided. Future investigation to better characterize *Borrelia burgdorferi*-associated fibrosing dermatoses and responsiveness to antibiotic therapy is worthwhile.

## Conflicts of interest

None disclosed.
